# Supplementary material for: Gene Electrotransfer into Mammalian Cells Using Commercial Cell Culture Inserts with Porous Substrate
Source: Pharmaceutics. 2022 Sep 16;14(9):1959. doi: 10.3390/pharmaceutics14091959 (PMC9506064; doi:10.3390/pharmaceutics14091959)
Supplement: Supplementary file 1 [file pharmaceutics-14-01959-s001.zip › pharmaceutics-1844418-supplementary.pdf]

Supplementary Information

Gene electrotransfer into mammalian cells using commercial cell culture inserts with porous substrate

Tina Vindiš,[1] Anja Blažič,[1] Diaa Khayyat,[1,2,3] Tjaša Potočnik,[1] Shaurya Sachdev[1] and Lea Rems[1,*]

[1] Faculty of Electrical Engineering, University of Ljubljana, Tržaška 25, 1000 Ljubljana, Slovenia
[2] Institute for Multiphase Processes, Leibniz University Hannover, An der Universität 1, 30823 Garbsen, Germany
[3] Lower Saxony Centre for Biomedical Engineering, Implant Research and Development, Stadtfelddamm 34, 30625 Hannover, Germany

* Correspondence: lea.rems@fe.uni-lj.si

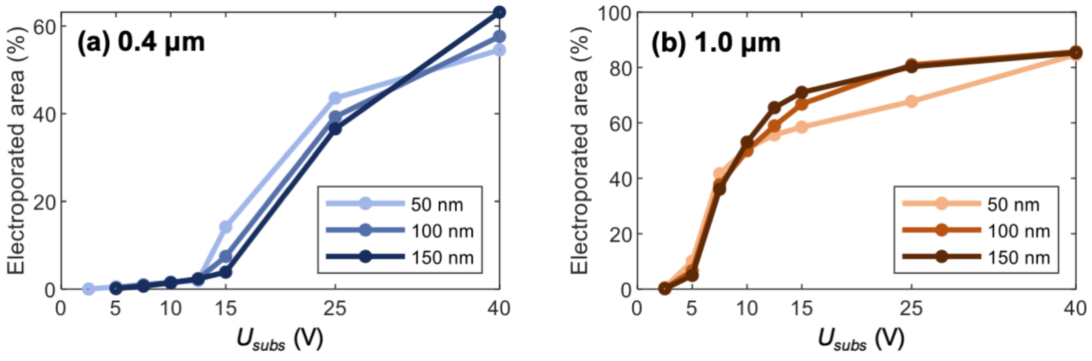

Fig. S1: Influence of the gap distance between the cell membrane and the porous substrate on relative electroporated area for different substrate voltages, U_{subs}. (a) Results for substrates with 0.4 μm pores. (b) Results for substrates with 1.0 μm pores. All calculations are for a cell with dimensions 40 μm × 15 μm.

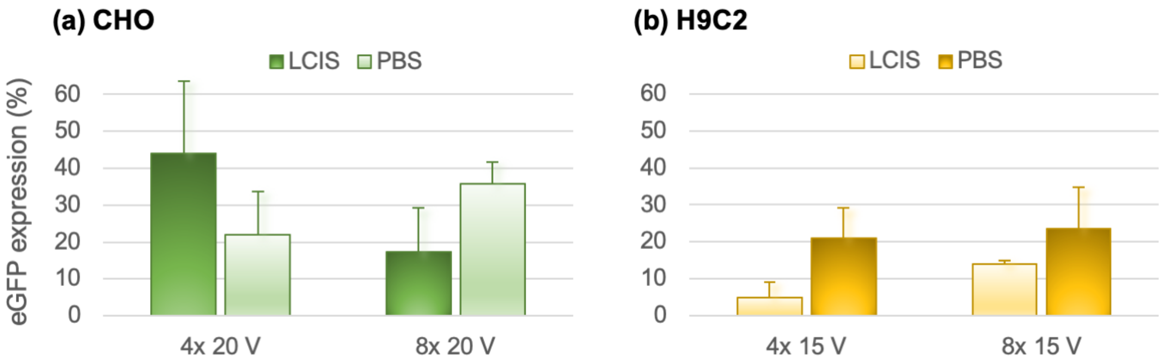

Fig. S2: Comparison between eGFP expression in CHO (a) and H9C2 (b) cell lines when 500 μg/ml plasmid is dissolved in LCIS or PBS. Percentage of eGFP positive cells is shown as mean ± s.d. of 2-4 experiments. For H9C2 cells the expression is better when plasmid is dissolved in PBS compared to LCIS; possibly the presence of Ca^{2+} in LCIS is problematic for H9C2 which are myoblasts derived from cardiomyocytes. For CHO the expression when plasmid is dissolved in LCIS and PBS depends on the number of pulses applied, possibly due to some overexpression toxicity. Statistical analysis based on Two Way ANOVA (solution type and pulse number as factors) with Holm-Sidak method for pairwise multiple comparison indicated no significant difference between LCIS and PBS for CHO cells, whereas this difference was significant for H9C2 cells (unadjusted P = 0.015).

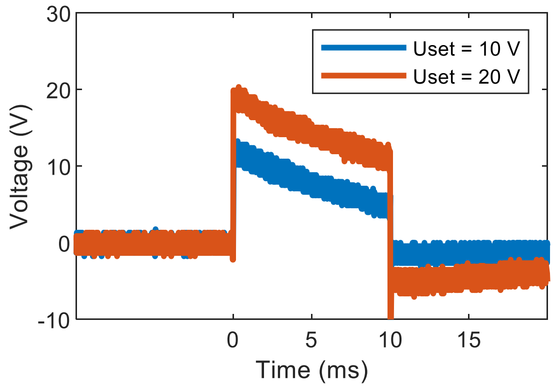

Fig. S3: Electric pulses with duration of 10 ms applied with the B10 electroporator. U_{set} is the voltage that was preset.

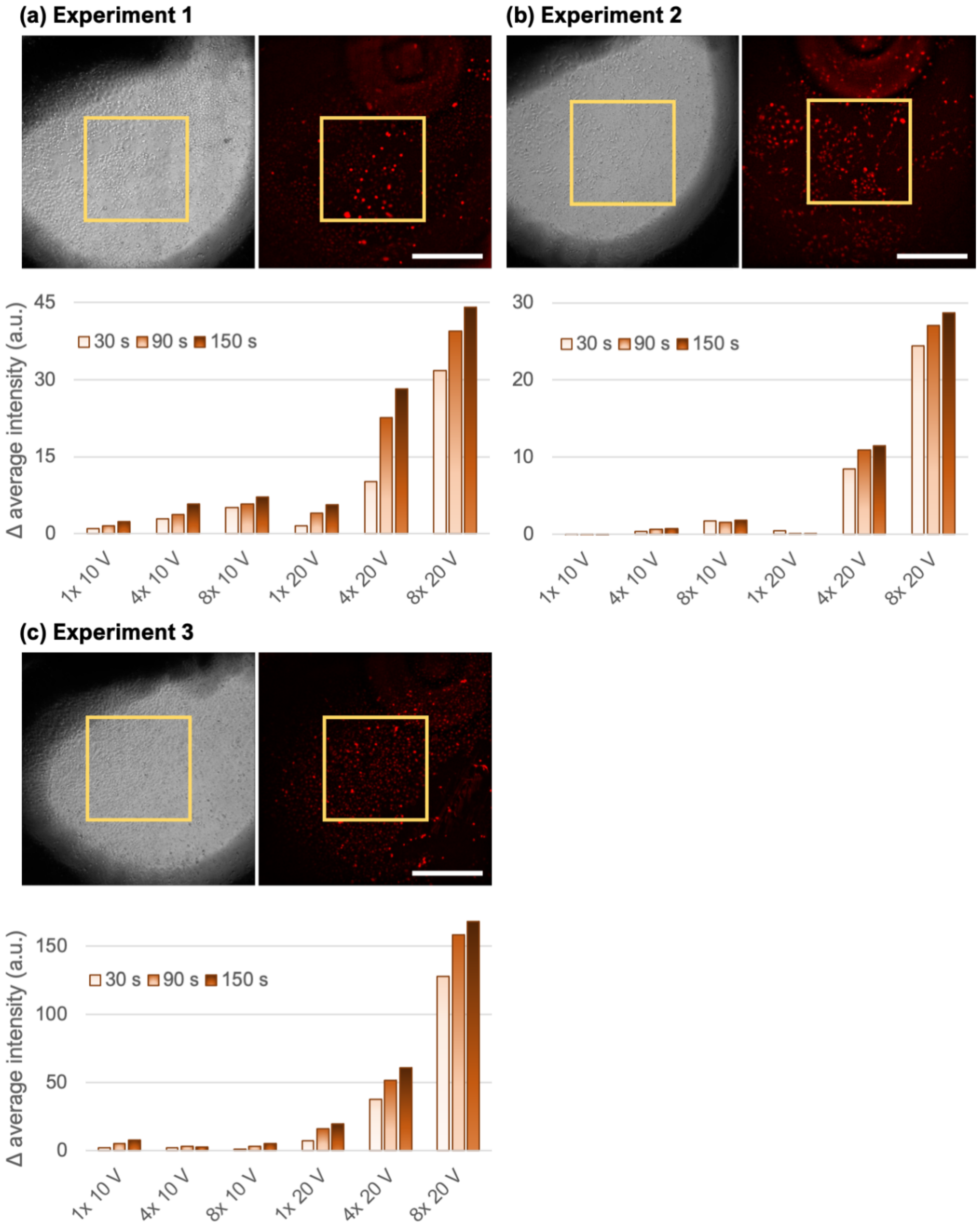

Fig. S4: Propidium iodide uptake measured in three independent experiments using substrates with 0.4 μm pores. Brightfield and fluorescence images are shown for 8x 20 V pulses. Scalebar 400 μm. Graphs show the change in average fluorescence intensity at indicated times after application of 10 ms pulses with selected amplitude and number. The change in intensity was determined by subtracting the average intensity within a region of interest (yellow rectangles) before pulse application from the average intensity at the given time after pulse application. The region of interest excluded the areas with the bottom electrode (black shades in brightfield images). Note that the absolute values of the change in fluorescence intensity are higher in Experiment 3 compared to Experiments 1 and 2, because the cells are more confluent in Experiment 3. However, all three experiments show qualitatively very similar results and confirm that pulse amplitude of 10–20 V is sufficient for electroporation. The graph in Fig. 4d of the main manuscript shows the mean and range of values form Experiment 1 and 2.

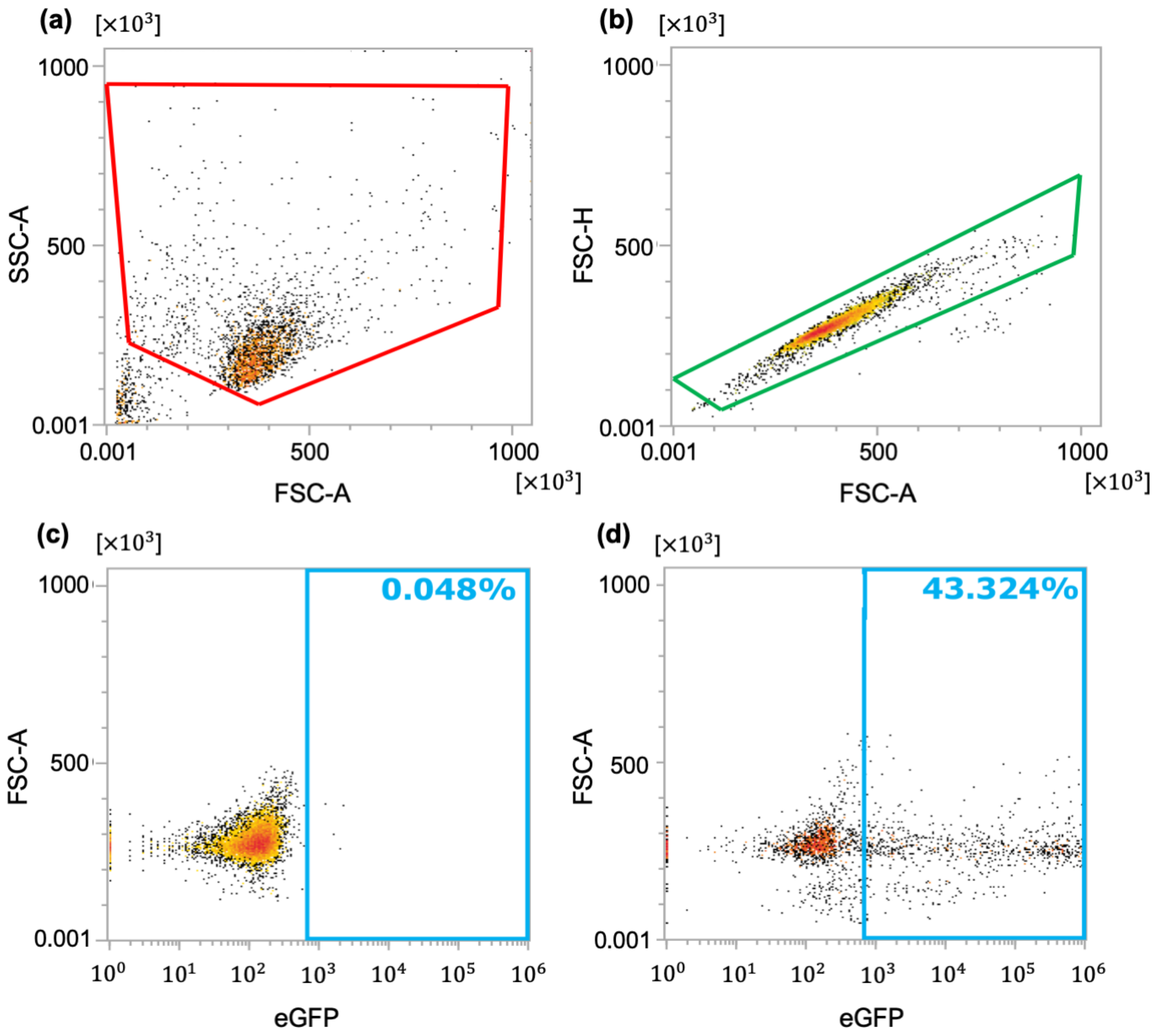

Fig. S5: Gating strategy in flow cytometry. (a) Region selecting live cells and removing cell debris. (b) Region removing cell clusters. (c,d) Gate for determining the percentage of transfected cells. Control sample is shown in (c) and electroporated/transfected sample is shown in (d).

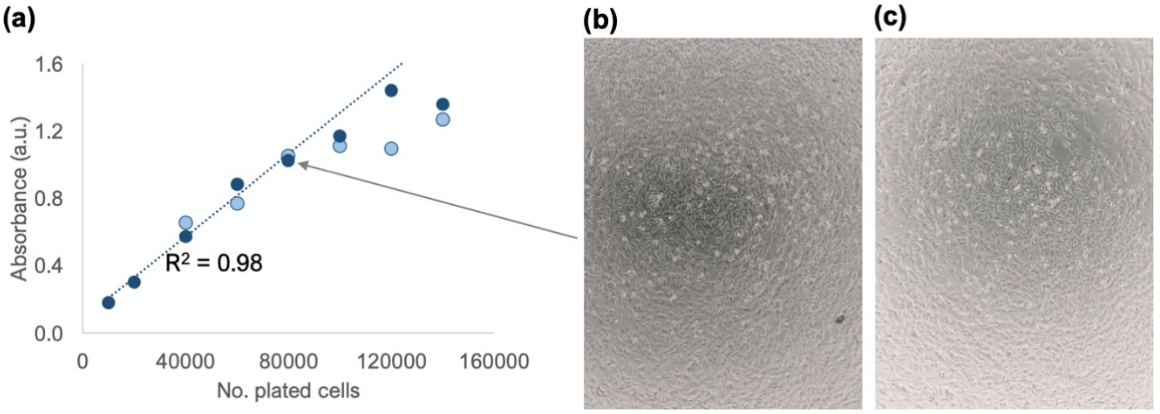

Fig. S6: Linear relationship between MTS absorbance and the number of CHO cells. Different numbers of cells were plated into inserts and the MTS assay was performed the next day after the cells have attached and spread over the surface. (a) Relationship between the measured absorbance of the MTS assay and the number of plated cells. Data points are from two independent experiments. The data shows that the relationship is linear up to

~80000 plated cells. Note that the number of plated cells in these measurements cannot be compared directly to the number of plated cells in cell viability experiments presented in the paper, since the cells for viability tests were grown for 3 days in total in the inserts. Since cell growth rate depends on the number of plated cells, we allowed the cells to grow for one day only when checking the linearity of the MTS assay. What we can compare with viability tests is the confluency of the cell monolayer. (b) Cell confluency corresponding to the data points indicated in the graph. (c) Maximum cell confluency when performing cell viability tests. This confluency was always well within the linear regime of the MTS assay. Images in (b) and (c) were captured at 10x objective magnification using inverted microscope EVOS XL Core Imaging System (ThermoFischer Scientific).

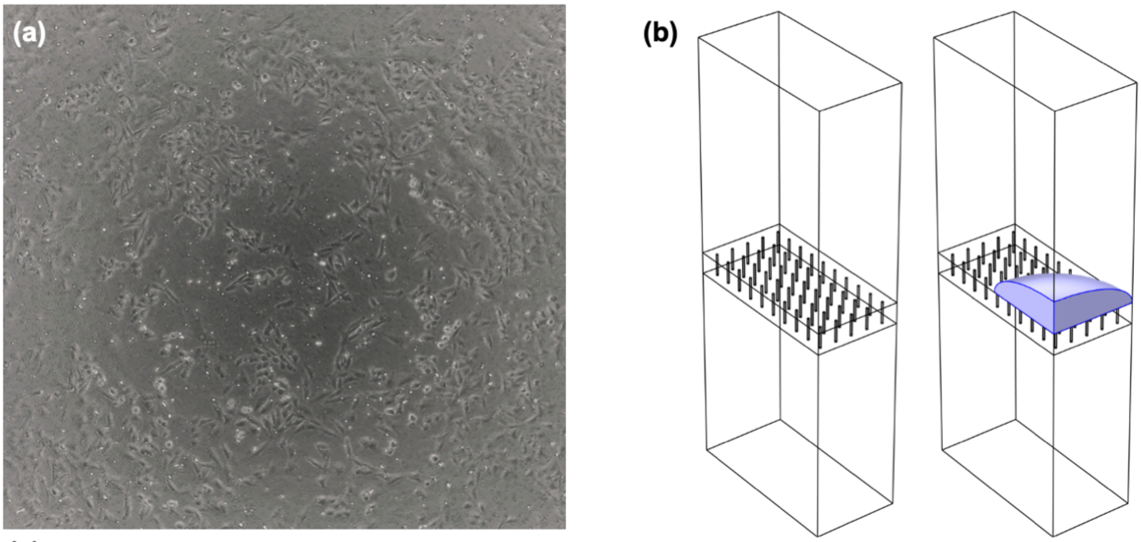

(c)

Parameter	Symbol	Substrate pore size	
		**0.4 µm**	**1.0 µm**
Conductance of the model without a cell	$G_{model\text{-}cell}$	8.3 mS	35 mS
Conductance of the model with a cell	$G_{model+cell}$	8.7 mS	44 mS
Model area	A_{model}	3200 µm^2	
Substrate area	A_{subs}	32 mm^2	
Substrate resistance of without cell	$R = (G_{model\text{-}cell} \cdot A_{subs}/A_{model})^{-1}$	**115 Ω**	**23 Ω**
Substrate resistance of with cell	$R = (G_{model+cell} \cdot A_{subs}/A_{model})^{-1}$	**121 Ω**	**29 Ω**

Fig. S7: Numerical results showing how the presence of cells influences the electrical resistance of the porous substrate. (a) Typical density of cells growing on a substrate, which was used for electroporation. This density allowed sufficient space for cells to divide and grow before analyzing the samples the next day, keeping the cells in exponential growth phase. The image shows CHO cells with 10x objective magnification. (b) Geometry of the model of the substrate with and without a cell on top of it. (c) Calculation of the substrate resistance for a model with and without a cell. The values in the first three columns are based on numerical calculations, similar to those in Fig. 2, but at conditions which do not lead to cell membrane electroporation. The difference in resistance with and without a cell is 6 Ω, which is a minor fraction of the resistance of the entire insert system (>90 Ω).

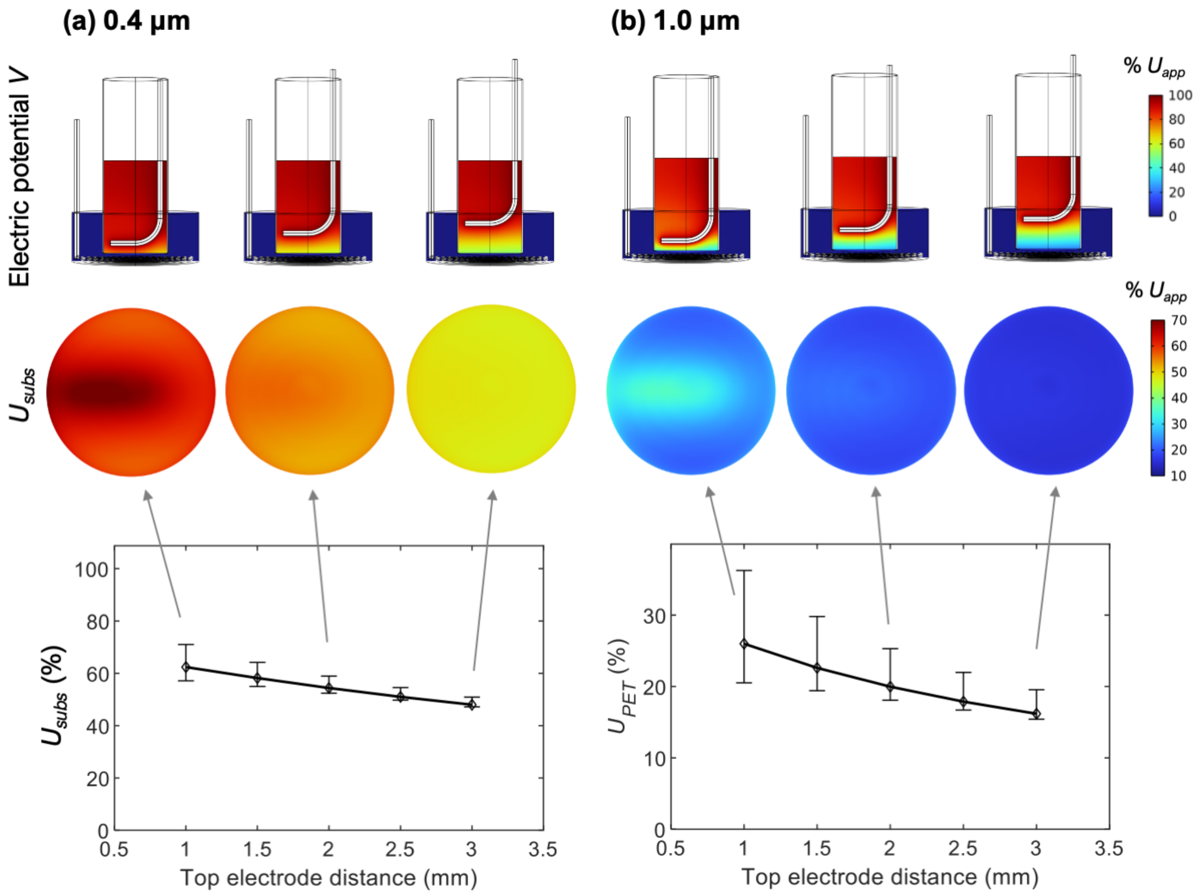

Fig. S8: Influence of the top electrode position on the substrate voltage, U_{subs}. (a) Results for 0.4 µm substrate pores. (b) Results for 1.0 µm substrate pores. In each panel the first row shows the electric potential distribution when the top electrode is positioned at 1 mm, 2 mm, and 3 mm from the substrate. The second row shows the corresponding distribution of U_{subs}. Note that the max value on the colorbar is different from Fig. 3. The graphs show the average, minimum, and maximum U_{subs} depending on the top electrode distance, expressed at the fraction of the applied voltage, U_{app}. The y-axis is scaled to 2x U_{subs} value when the top electrode is positioned 2 µm above the substrate. Note that in relative terms, variations in top electrode positions have considerably greater influence on U_{subs} when using 1.0 µm substrate pores.

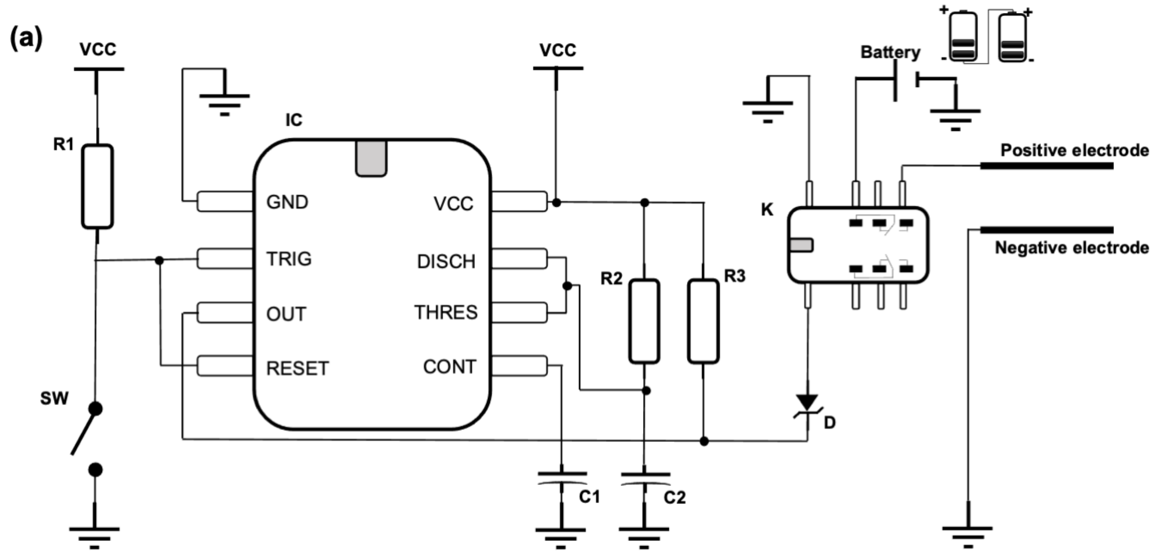

(b)

Name	Symbol	Value
button	SW	
precision timer NA555	IC	
relay G5V-2-H1	K	
Zener diode	D	3 V
resistor	R1	10 kΩ
resistor	R2	1.3 MΩ
resistor	R3	1 kΩ
capacitor	C	10 nF
battery (chip supply)	VCC	9 V
battery (output supply)	Battery	2x 9 V

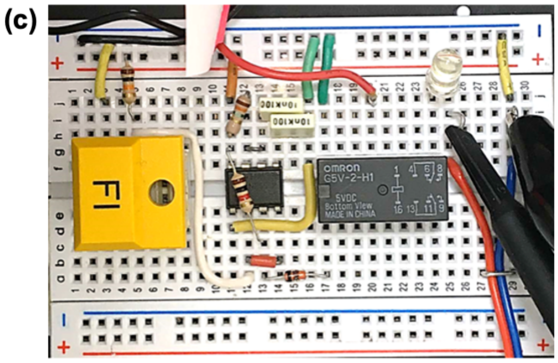

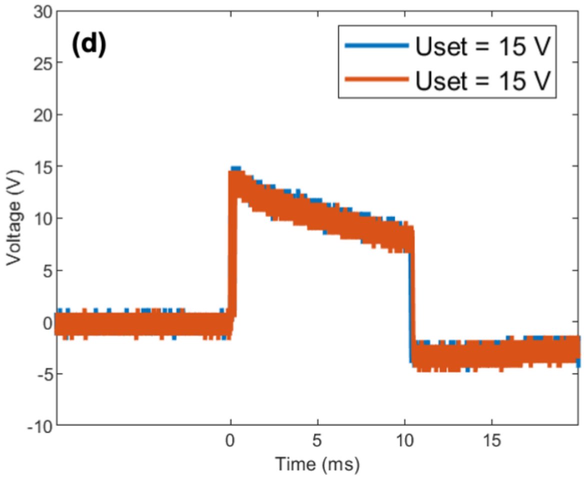

Figure S9. Simple electrical circuit powered by 9 V batteries for generating the pulses. (a) Electrical scheme. (b) List of elements. (c) Picture of the electrical circuit assembled on protoboard. (d) Time course of two independently generated pulses, overlayed. The pulses are reproducible and resemble those generated with B10 electroporator (Suppl. Fig. S3). As proof of concept, we transfected CHO and H9C2 cells using 500 µg/ml concentration with this electrical circuit and obtained 32% and 17% transfected cells, respectively (n = 1), which is similar to the transfection efficiency obtained with B10 electroporator (Fig. 7).
